# Hornerin deposits in neuronal intranuclear inclusion disease: direct identification of proteins with compositionally biased regions in inclusions

**DOI:** 10.1186/s40478-022-01333-8

**Published:** 2022-03-04

**Authors:** Hongsun Park, Tomoyuki Yamanaka, Yumiko Toyama, Atsushi Fujita, Hiroshi Doi, Takashi Nirasawa, Shigeo Murayama, Naomichi Matsumoto, Tomomi Shimogori, Masaya Ikegawa, Matti J. Haltia, Nobuyuki Nukina

**Affiliations:** 1 Laboratory of Structural Neuropathology, Doshisha University Graduate School of Brain Science, 1-3 Miyakodanitatara, Kyotanabe-shi, Kyoto, 610-0394 Japan; 2 Department of Neuroscience of Disease, Brain Research Institute, Niigata University, Niigata, Japan; 3 Department of Life and Medical Systems, Doshisha University, Kyoto, Japan; 4 Department of Human Genetics, Yokohama City University Graduate School of Medicine, Yokohama, Japan; 5 Department of Neurology and Stroke Medicine, Yokohama City University Graduate School of Medicine, Yokohama, Japan; 6 Bruker Japan K.K., Yokohama, Japan; 7 The Brain Bank for Aging Research, Tokyo Metropolitan Geriatric Hospital and Institute of Gerontology, Tokyo, Japan; 8 Molecular Mechanisms of Brain Development, RIKEN Center for Brain Science, Saitama, Japan; 9 Department of Pathology, Faculty of Medicine, University of Helsinki, Helsinki, Finland; 10 Laboratory for Structural Neuropathology, RIKEN Brain Science Institute, Saitama, Japan

**Keywords:** NIID, Neuronal intranuclear inclusion, Hornerin, Amino acid analysis, Compositionally biased region, Low sequence complexity

## Abstract

Neuronal intranuclear inclusion disease (NIID) is a neurodegenerative disorder, characterized by the presence of eosinophilic inclusions (NIIs) within nuclei of central and peripheral nervous system cells. This study aims to identify the components of NIIs, which have been difficult to analyze directly due to their insolubility. In order to establish a method to directly identify the components of NIIs, we first analyzed the huntingtin inclusion-rich fraction obtained from the brains of Huntington disease model mice. Although the sequence with expanded polyglutamine could not be identified by liquid-chromatography mass spectrometry, amino acid analysis revealed that glutamine of the huntingtin inclusion-rich fraction increased significantly. This is compatible with the calculated amino acid content of the transgene product. Therefore, we applied this method to analyze the NIIs of diseased human brains, which may have proteins with compositionally biased regions, and identified a serine-rich protein called hornerin. Since the analyzed NII-rich fraction was also serine-rich, we suggested hornerin as a major component of the NIIs. A specific distribution of hornerin in NIID was also investigated by Matrix-assisted laser desorption/ionization imaging mass spectrometry and immunofluorescence. Finally, we confirmed a variant of hornerin by whole-exome sequencing and DNA sequencing. This study suggests that hornerin may be related to the pathological process of this NIID, and the direct analysis of NIIs, especially by amino acid analysis using the NII-rich fractions, would contribute to a deeper understanding of the disease pathogenesis.

**Supplementary Information:**

The online version contains supplementary material available at 10.1186/s40478-022-01333-8.

## Introduction

Neuronal intranuclear inclusion disease (NIID) is a slowly progressive neurodegenerative disorder, characterized pathologically by eosinophilic inclusions within the nuclei of central, peripheral and autonomic nervous system cells. The first case of the disease was described in 1968 [1], and since then, a number of cases have been reported, based on autopsy findings [2–4]. Antemortem diagnosis has been difficult because NIID shows variable clinical manifestations, including cognitive impairment, parkinsonism and neuropathy at varying ages of onset, ranging from infancy to late adult age [5]. Recently, NIID has been considered to be not only clinically but also genetically heterogeneous [6].

Neuronal intranuclear inclusions (NIIs) are thought to be formed due to an excessive accumulation of proteins within the nucleus [7]. Apart from NIID, they may be associated with various other neurodegenerative diseases, caused by the expansion of trinucleotide repeats [8]. For example, nine neurodegenerative diseases, including Huntington disease (HD), spinal and bulbar muscular atrophy, dentatorubral-pallidoluysian atrophy and six autosomal dominant forms of spinocerebellar ataxia (SCA1, 2, 3, 6, 7, and 17), are caused by expansions of CAG repeats, encoding expanded polyglutamine tracts, and have NIIs containing the respective pathogenic protein [9]. Thus, the inclusions of these diseases are supposed to be composed of the proteins with low complexity regions (LCRs). Even cytoplasmic inclusions can be formed by accumulation of misfolded proteins, such as ⍺-synuclein in Parkinson disease (PD) or tau protein in Alzheimer disease (AD), which are thought to contain LCRs in their sequences. Such proteins are known as intrinsically disordered proteins (IDPs), characterized by the presence of intrinsically disordered regions [10]. Unlike folded domains, intrinsically disordered regions do not adopt stable secondary structures, and the LCRs exhibit compositional bias towards a small set of amino acids [11], causing proteins to misfold. Other expanded repeats can be translated into proteins containing a pathogenic stretch of repeated amino acids by a novel mechanism called repeat-associated non-AUG (RAN) translation [12], and these proteins could contain compositionally biased amino acids.

In 2011, Sone et al. [13] reported that skin biopsies are useful for the antemortem diagnosis of NIID, and skin biopsies have contributed to an increased number of patients diagnosed with NIID. Later, the disease of a number of NIID patients diagnosed by skin biopsy was shown to be caused by the mutant *NOTCH2NLC* gene with expanded GGC or CGG repeats [14, 15]. The mutant *NOTCH2NLC* gene expressing 69 GGC repeats is translated into NOTCH2NLC-polyglycine protein [16]. Furthermore, GGC repeats of NOTCH2NLC (N2C) protein have been thought to be embedded in a small upstream open reading frame (uORF) located ahead of the main N2C ORF, encoding a small protein, uN2C, with an expanded polyglycine stretch (uN2CpolyG) in this form of NIID [17].

Unlike these NIID cases, which were mostly found in people of Asian origin [6], the NIID brain sample used in this study showed different features. This unique NIID case [2], one of concordant twins, had juvenile-onset without any identified expanded repeats such as CAG or GGC, and the NIIs found in this case were autofluorescent and showed a fibrillar ultrastructure [2, 6]. In addition, an infantile NIID case has been reported with no expanded repeats. Skin biopsy had not been helpful [18]. This case provides additional evidence suggesting genetic heterogeneity of NIID and implies that analyses of *NOTCH2NLC* GGC repeats and/or skin biopsy testing may not reliably diagnose the disease in suspected patients. Although studies have been focused mainly on the genes related to NIIs pathology, little remains known about the components of NIIs and the pathogenesis of NIID. However, because the deposited protein is not always a mutant protein, as shown by e.g. Aβ deposition in presenilin mutations, it is important to identify the deposited protein to reveal the disease cascade.

It has been considered that the composition of NIIs can be distinct in different diseases, but this has been difficult to analyze directly, due to the insolubility of the proteins. In this study, using an NIID brain sample [2], we investigated the components of NIIs directly by several proteomic analyses, including liquid-chromatography mass spectrometry (LC–MS/MS), Matrix-assisted laser desorption/ionization imaging mass spectrometry (MALDI-IMS), shotgun proteomics and amino acid analysis, and finally identified hornerin as the main component of the NIIs in this case.

## Materials and methods

### HD190QG transgenic mice

All surgical procedures and experiments were approved by the Doshisha university ethics committee and the RIKEN ethics committee. All animal experiments were performed in accordance with the guidelines approved by the Doshisha university and RIKEN Animal Experiments Committee. All experiments were carried out in accordance with the approved ethical guidelines and regulations. Huntington disease model mice expressing the first exon of human huntingtin with an expanded polyglutamine fused with an enhanced green fluorescent protein (EGFP) under the control of the human *huntingtin* promoter were generated as described previously [19]. 24-week-old HD190QG male mice and the wild type (WT) littermates were used in this study.

### Human brain samples

The NIID brain sample for this study was reported previously [2]. The brain was removed 3 h postmortem and kept at − 80 °C. Control 1 (55 years old, male) and Control 2 (50 years old, female) brains were approved by Doshisha university. Human cortical specimens for imaging mass spectrometry were also obtained from the Brain Bank at Tokyo Metropolitan Institute of Gerontology. Brains were removed, processed and stored at − 80 °C within 8 h postmortem. Patients were placed in a cold room (4 °C) within 2 h after death. All brains registered at the Brain Bank came with written informed consents for their use in medical research from the patients or their families. Brain specimens were taken from the occipital cortex of AD and age-matched control patients as well as NIID. This study was approved by the ethics committee at Doshisha University and Tokyo Metropolitan Geriatric Hospital.

### Isolation of NIIs

Mouse brains were homogenized in 9% sucrose in a homogenization buffer [9% sucrose in 2 mM EDTA (pH 8.0)/25 mM Tris–HCl (pH 8.0)/5 mM DL-Dithiothreitol (DTT)] with a protease inhibitor, cOmplete™ (Roche), using Potter–Elvehjem Tissue Grinders. Brain lysate was then centrifuged at 12,000×*g* for 10 min at 4 °C, and the pellet (P1) was resuspended in a homogenization buffer without sucrose. The solution was filtered with a 40 µm cell strainer (Falcon), and the flow-through was collected as P1 solution. The P1 solution (1 mg/ml) was then treated with 1% sarkosyl solution at room temperature for 1 h while rotating. The solution was ultracentrifuged at 153,725×*g* (50,000 rpm; Optima™ MAX-XP Ultracentrifuge, TLA-55 rotor, Beckman) for 30 min at 4 °C. The pellet (P2) was washed with 50 mM Tris–HCl (pH 7.6) and ultracentrifuged again at 153,725×*g* for 30 min at 4 °C. After resuspending the pellet (P3) with 50 mM Tris–HCl (pH 7.6) using a glass homogenizer, P3 solution was treated with nuclease solution [Micrococcal nuclease (1:400, Thermo Scientific), 5 mM CaCl_2_, 50 mM Tris–HCl (pH7.6)] for 30 min at 37 °C until the nucleic acids were digested. The reaction was stopped by adding 20 mM EGTA (pH 7.6), and the solution was ultracentrifuged at 153,725×*g* for 30 min at 4 °C. After the nuclease treatment, the pellet (P4) was resuspended with 200 µl of homogenization buffer, treated with 2% sodium dodecyl sulfate (SDS)/10 mM DTT at 100 °C for 5 min, followed by ultracentrifuging at 153,725×*g* for 30 min at 20 °C. The supernatant was collected as the SDS-soluble fraction. The SDS-insoluble pellet was treated with 70% formic acid (FA), sonicated for 30 s twice (Bioruptor sonicator, BMBio) and incubated at 37 °C for 1 h. After evaporating in a vacuum, the remaining proteins, i.e., the FA-treated fraction, were considered to include the components of NIIs.

The NII-rich fractions of human brains were obtained directly from NIID and the control brains using the same protocol of mouse brain nuclei fractionation but with an additional SDS-wash after treating the resuspended P4 fraction with 2% SDS/10 mM DTT to remove the remaining SDS-soluble proteins in the tube.

### Antibodies

We used the following primary antibodies: EM48 (1:500 for Western blot (WB), 1:1,000 for immunofluorescence (IF), MAB5374, Chemicon), anti-GFP (1:1,000 for WB and IF, 598, MBL), anti-ubiquitin (1:1,000 for WB and IF, MAB1510, Merck Millipore) and anti-hornerin (1:500 for WB, HPA-31469, Sigma-Aldrich) antibodies. Anti-HRNR (HP) antibody used for IF (1:1000) was designed to detect human HRNR_968-1349_ (made in our laboratory with CosmoBio).

The following secondary antibodies were used: HRP-conjugated mouse IgG (1:2,000 for WB, GE healthcare), HRP-conjugated rabbit IgG (1:2,000 for WB, GE healthcare), Alexa Flour® 546 anti-mouse IgG (1:300 for IF, Life technologies), Alexa Fluor® 594 anti-rabbit IgG (1:300 for IF, Invitrogen), Alexa Fluor® 647 anti-mouse IgG (1:300 for IF, Invitrogen) and Alexa Fluor® 647 anti-rabbit IgG (1:300 for IF, Life technologies) antibodies.

### Immunofluorescence

The smears of each fraction were dried overnight on a slide glass and blocked in 500 µl of blocking buffer containing 0.1% TritonX-20 and 2.5% goat serum in Tris Buffered Saline with 0.1% Tween 20 (TBST) for 30 min at room temperature. After the incubation with primary antibodies diluted in 0.1% bovine serum albumin (BSA)/TBST overnight at 4 °C., the samples were incubated with fluorescent secondary antibodies at room temperature for 2 h.

The brain sections (10 µm-thick) were washed in phosphate buffered saline (PBS) for 10 min and fixed with 4% paraformaldehyde in phosphate buffered saline (PFA/PBS) for 5 min at room temperature. After heat activation in 0.1% citrate buffer (pH 6.0) at 121 °C for 5 min, the brain sections were cooled down on ice for 10 min, washed with MilliQ and TBST sequentially and blocked in 0.1% BSA/TBST at room temperature for 1 h. After the primary antibody incubation diluted in TBST overnight at 4 °C, the brain sections were incubated with fluorescent secondary antibodies at room temperature for 2 h.

The samples were mounted with VECTASHIELD® mounting medium for fluorescence with DAPI (H-1200, Vector Laboratories), and the images were taken with BIOREVO BZ-9000 (KEYENCE).

### Western blot

Equal amounts of P1 solution, SDS-soluble and FA-treated fractions were re-solubilized in SDS containing Laemmli buffer. The proteins were separated in a 5–20% SDS–polyacrylamide gel (e-PAGEL®, E-T520L, ATTO) and transferred to polyvinylidene difluoride membranes (Immobilon®-P Transfer Membrane, Merck Millipore). The membranes were blocked with 2.5% goat serum/TBST and incubated with primary antibodies diluted with 2.5% goat serum/TBST at 4 °C, overnight. The membranes were washed in TBST for 10 min twice and incubated in secondary antibodies diluted with 2.5% goat serum/TBST for 1 h, at room temperature. The immunoreactive proteins were visualized with the application of the substrate for enhanced chemiluminescence (Luminata, Merck Millipore). The signals were acquired using ImageQuant LAS-4000 (GE Healthcare).

### Protein digestion and LC–MS/MS

Protein tryptic digestion was performed with the Filter-aided Sample Preparation (FASP) method [20]. Samples were mixed with UA solution (8 M Urea in 100 mM Tris–HCl, pH 8.0) in filter units (molecular weight cut-off 30 kDa, PT-1007, Aproscience) and centrifuged at 14,000×*g* for 15 min at room temperature, followed by the addition of IAA solution (0.05 M iodoacetamide in UA). The samples were then incubated for 20 min in the dark at room temperature and filtered by centrifuging them at 14,000×*g* for 10 min. After the centrifugation, 100 µl of UA was added to the filter, followed by centrifugation at 14,000×*g* for 15 min. This step was repeated twice. The proteins trapped on the filters were washed with AmBic solution (50 mM ammonium bicarbonate in MilliQ) three times, and the residual proteins were enzymatically digested by 40 µl of 0.2 μg/μl modified trypsin (V511A, Promega) in AmBic solution (enzyme to protein ratio 1: 100) overnight at 37 °C. The digested proteins were collected by centrifuging at 14,000×*g* for 10 min after adding a total of 80 µl AmBic solution and 50 µl of 0.5 M NaCl to the filter units. The filtrates were then applied to the LC–MS/MS system.

In-gel digestion was performed using the Thermo Scientific™ In-Gel Tryptic Digestion Kit (89,871, ThermoScientific) in order to obtain and identify peptides of interest stained by Coomassie Brilliant Blue (CBB). The digested proteins were analyzed by LC–MS/MS.

To obtain MS/MS spectrum data of the peptides, the digested peptides were separated by EASY-nLC 1000 (Thermo Fisher Scientific) and ionized with nano-ESI, followed by analyzation using a QExactive hybrid quadrupole-orbitrap mass spectrometer (Thermo Fisher Scientific). Based on this peptide information, proteins were identified using Proteome Discoverer version 2.4 (PD2.4, Thermo Fisher Scientific) with the MASCOT search engine software (Matrix Science) [21].

### MALDI-IMS and LC-TIMS-MS/MS

Autopsied brains were kept in a deep freezer before experimentation began. 10 µm cryosections were cut and transferred to Indium-Tin-Oxide (ITO) coated glass slides. For proteomic imaging, slides were washed with 70% to 100% EtOH, Carnoy’s solution (EtOH: Chloroform: Acetic acid = 6: 3: 1), and 0.1% Trifluoroacetic acid (TFA). Tissue sections were sprayed with CHCA (α-cyano-4-hydroxycinnamic acid, 10 mg/ml) in 70% acetonitrile (ACN) containing 1% TFA using an automated sprayer (TM-Sprayer™, HTX Technologies, Carrboro, NC, USA). Mass spectra were measured by rapiflex tissuetyper (Bruker Daltonics) with a spatial resolution of 20 µm. For Protein ID experiments, trypsin solution (25 ng/µl, in 20 mM NH4HCO3aq, pH 7.5–8.5) was sprayed at room temperature and incubated for 2 h, at 50 °C. Peptide extraction from brain tissue samples was performed with 25–50 µl of 0.1% TFA onto the tissues and peptide extracts were stored at − 80 °C for LC-TIMS-MS/MS analysis (timsTOF Pro, Bruker Daltonics). MALDI-IMS data were obtained and analyzed using the flexImaging 5.0 and SCiLS Lab 2018b. For proteomic analysis, PEAKS Studio 8.5, ProteinScape and MASCOT software were employed [22].

### Amino acid analysis

SDS-insoluble fractions of HD190QG mouse brains and human brains were hydrolyzed in 6 M HCl and 4 N Methanesulfonic acid (MSA), respectively. After the hydrolysis at 110 °C for 20 h, 4 M NaOH was added to neutralize the MSA. The derivatives were detected using the ninhydrin method to prevent 3-(2-aminoethyl) indole from disturbing the AQC-amino acid chromatogram. The products were detected by High Speed Amino Acid Analyzer, L-8900 (Hitachi High-Technologies). Chromatograms were not normalized in the MSA hydrolysis.

### Whole-exome sequencing and repeat analysis of *NOTCH2NLC*

To identify the pathogenic variant, we performed whole-exome sequencing of　genomic DNA extracted from the patient’s brain. Genomic DNA was processed using the SureSelect Human All Exon Kit v5 (Agilent Technologies, Santa Clara, CA) and sequenced on an Illumina HiSeq 2500 with 101-bp paired-end reads. Alignment, variant calling and annotation were performed using Novoalign (http://www.novocraft.com/), Picard (http://picard.sourceforge.net/), Genome Analysis Toolkit (https://www.broadinstitute.org/gatk/index.php) and ANNOVAR (http://www.openbioinformatics.org/annovar/) as described previously [23]. Single nucleotide variants (SNVs) and insertion/deletions (indels) were extracted based on information regarding rare variants with a minor allele frequency < 1% in dbSNP137, located in exons or splice sites (within 10 bp of the boundaries).

Repeat-primed PCR for detecting GGC expansions in *NOTCH2NLC* and fluorescence amplicon length analysis for determining the GGC expansion length in *NOTCH2NLC* were performed as previously described [14]. Library preparation for long-read sequencing was performed using DNA ligation kit (SQK-LSK109, Oxford Nanopore Technologies, Oxford, UK) after shearing genomic DNA with g-TUBE (Covaris, Woburn, MA, USA), according to the manufacturer’s instructions. This library was sequenced on PromethION with a PRO-002 flow cell (R9.4.1, Oxford Nanopore Technologies). Base-calling and fastq file conversion were performed with MinKNOW v1.14.0 (Oxford Nanopore Technologies). Reads were aligned to the human reference genome hg38 using LAST (https://gitlab.com/mcfrith/last) [24], and the tandem repeat number was evaluated using tandem-genotypes v1.1.0 (https://github.com/mcfrith/tandem-genotypes) [25].

### DNA sequencing

Genomic DNA of NIID, Control 1 and Control 2 brains as well as HeLa and HEK293T cells were prepared as templates for polymerase chain reaction (PCR) using two primers, CATCTAGGAGCGAACAACATGG and ATAGCCAGAAGACTGACTTGAGC, to amplify 446 bp hornerin sequence including the variant site. The PCR products were then subjected to electrophoresis with a 2% agarose gel, and the bands were excised and purified using Wizard® SV Gel and PCR Clean-Up System. The purified PCR product was treated with BigDye® Terminator v3.1 Cycle Sequencing Kit (Thermo Fisher Scientific), followed by ethanol precipitation. The resulting PCR products were dissolved in highly deionized formamide and sequenced.

## Results

### The NII-rich fraction was obtained from HD model mouse brains

**Fig. 1:**
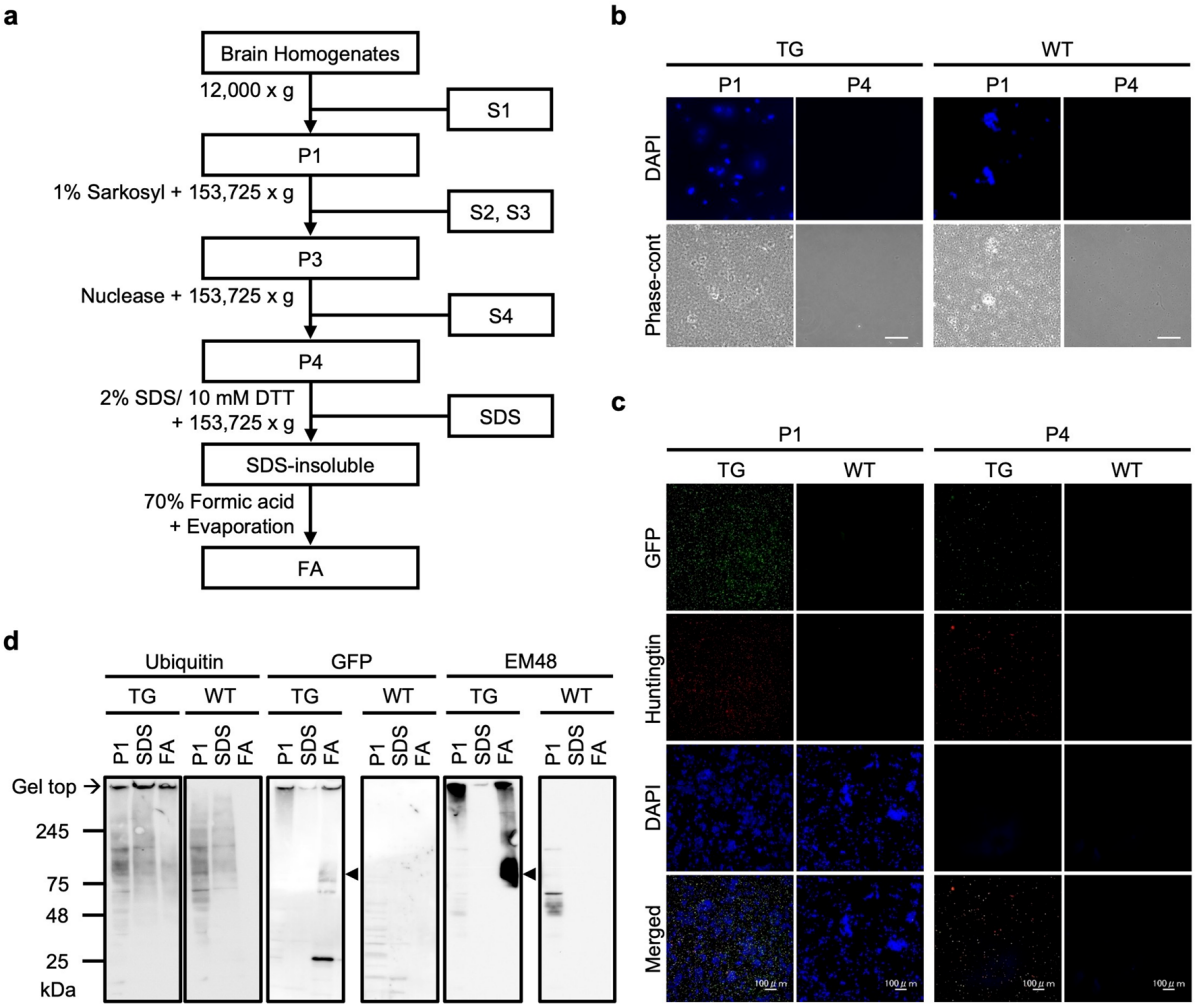
The NII-rich fraction was isolated from HD190QG mouse brains. **a** HD190QG mouse brains were subjected to nuclei fractionation. After the 2% SDS/10 mM DTT treatment, the SDS-insoluble fraction (SDS-insoluble) and the SDS-soluble fraction (SDS) were obtained. By treating the SDS-insoluble fraction with 70% FA, NIIs was obtained and used for further experiments (FA). **b** Phase-contrast microscopy of P1 and P4 fractions stained with DAPI. Scale bars = 50 µm. **c** IF staining of the P1 and P4 fractions stained with specific antibodies to GFP and huntingtin (EM48). Scale bars = 100 µm. **d** Western blotting of the P1, SDS and FA fractions stained with specific antibodies to ubiquitin, GFP and huntingtin (EM48). The broad bands indicated by arrowheads are nHTT-EGFP. TG refers to HD190QG transgenic mouse samples, and WT refers to wild type

To establish a novel method for directly identifying the NII components, we first examined whether glutamine increased in huntingtin inclusions obtained from the brains of HD model mice. We used HD190QG transgenic mice which express the first exon of huntingtin (N-terminal huntingtin: nHTT) with an additional expanded polyglutamine fused with an EGFP (nHTT-EGFP) under the control of the human *huntingtin* promoter [19, 26]. NIIs were isolated from the brains of 24-week-old HD190QG transgenic mice (Fig. 1a). The nuclear fractions from brain homogenates were treated with 1% sarkosyl solution and nuclease, followed by ultracentrifugation, in order to solubilize other proteins from the inclusion bodies as well as to degrade nucleic acids in the fraction [27]. By observing the smears of P1 and P4 fractions stained with DAPI, we confirmed that the chromatins in the P4 fractions were well degraded (Fig. 1b). To investigate whether nHTT inclusions remained in P4 after certain treatments, immunofluorescence staining was performed with EM48, an anti-HTT antibody (Fig. 1c). EM48-positive GFP signals were detected both in P1 and P4 of HD190QG transgenic mouse brains (TG), while no signals were detected in WT, indicating that nHTT inclusions were present in the P4 fraction. After the 2% SDS/10 mM DTT and 70% FA treatment to P4, the solubilized fractions were considered to include the components of NIIs. Western blot was performed with the P1 and FA-treated fractions to confirm that the isolation of NIIs was successful. The SDS-soluble fraction was examined in order to compare it with the SDS-insoluble fractions that were later treated by FA (Fig. 1d). In the fractions obtained from the HD190QG brains, the ubiquitin antibody-positive signals were observed at the gel top of FA, while no signals were observed in the WT fractions. Since ubiquitin becomes covalently bonded to many types of pathological inclusions, such as neurofibrillary tangles in AD [28] and Lewy bodies in PD [29], the signals detected at the gel top in TG indicate that the fractions include ubiquitinated inclusions. To make sure that the nHTT inclusions from HD190QG were included in SDS-insoluble (FA-treated) fractions, the fractions were immunostained with an anti-GFP antibody and EM48. In TG, anti-GFP antibody-positive and EM48-positive signals were detected not only at the gel top, but also inside the gel more strongly, indicating that solubilized monomers including human nHTT and EGFP with the size of around 75 kDa were detected. Using this FA-treated fraction, the components of NIIs obtained from HD190QG including nHTT inclusions was investigated via LC–MS/MS analysis.

### Huntingtin and EGFP were identified in NIIs of HD190QG by LC–MS/MS

**Fig. 2:**
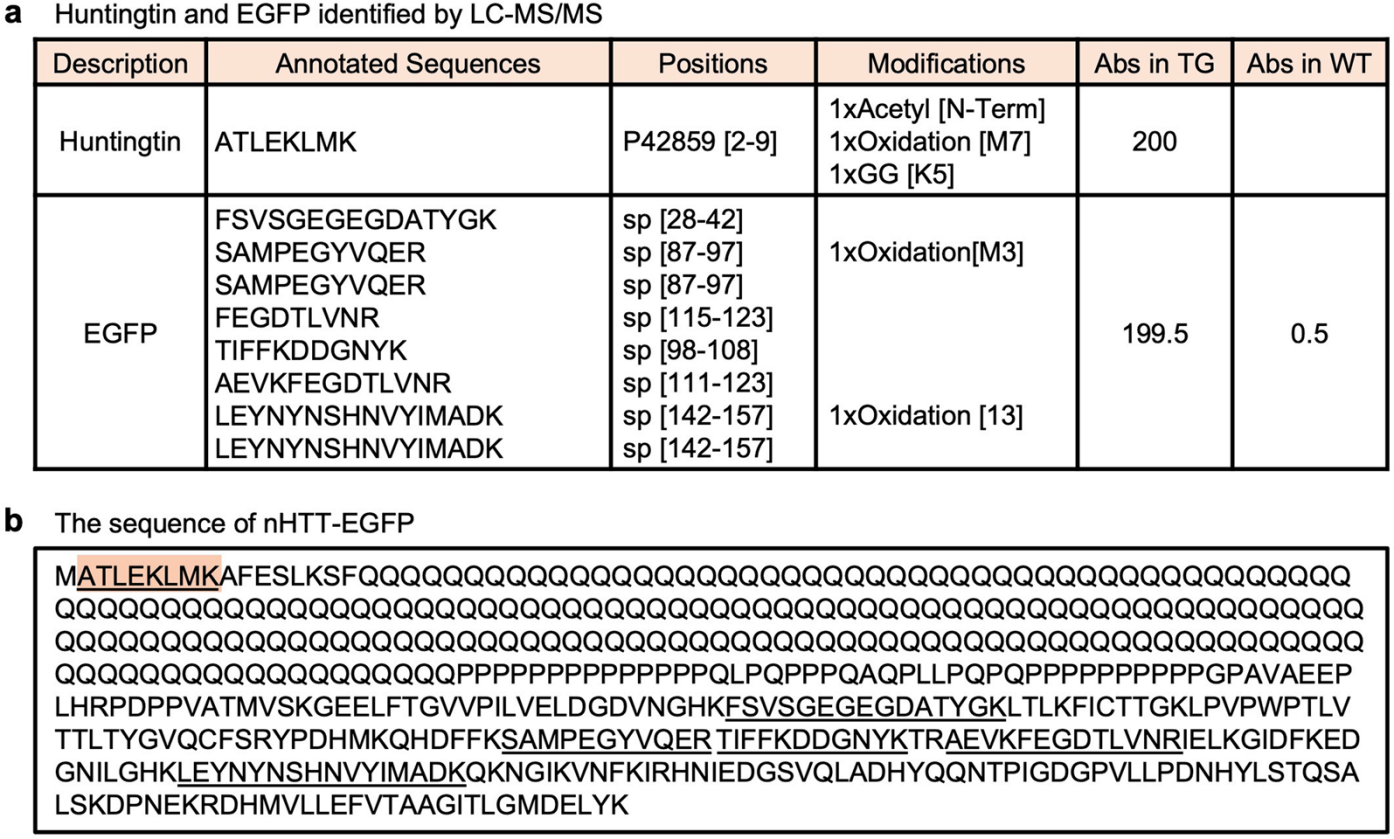
The peptides of huntingtin and EGFP were identified in the NIIs obtained from HD190QG mouse brains. **a** Huntingtin and EGFP were found only in the NIIs obtained from HD190QG transgenic mouse brains by LC–MS/MS. A modification 1xGG [K5] indicates that lysine 6 of huntingtin is ubiquitinated in insoluble nHTT inclusions. TG refers to HD190QG transgenic mouse samples, and WT refers to wild type. Abs refers to abundances. **b** The sequence of nHTT-EGFP with expanded polyglutamine (190Q) is shown, and the peptides identified by LC–MS/MS are underlined. The highlighted sequence was found both in mouse and human huntingtin

As the FA-treated fraction is rich in NIIs directly obtained from HD190QG, we assumed that human huntingtin and EGFP would be identified by LC–MS/MS. The trypsin digested FA-treated fractions of HD190QG and WT were subjected to LC–MS/MS. By comparing the relative scaled abundances between HD190QG and WT, 15 proteins including mouse huntingtin and EGFP were identified in NIIs of HD190QG (Additional file 1, Fig. 2a). Since human huntingtin was used for generating the HD190QG transgenic mice, we compared the sequences of mouse huntingtin and human huntingtin and found that both proteins shared the same sequence of the identified peptide. For EGFP, the identified sequences were found in the sequence of pEGFP-N1 vector, which was used for generating the HD190QG transgenic mice [26], indicating that the main components of nHTT inclusions were identified by LC–MS/MS (Fig. 2b). Next, we investigated the ubiquitination sites of nHTT in the NIIs. Previously, several ubiquitination sites of huntingtin were identified, and K6 and K9 were reported as the main sites for ubiquitination of the mutant huntingtin [30–32]. As ubiquitination occurs by forming an isopeptide bond between the C-terminal glycine of ubiquitin and a lysine of the target protein, we investigated whether there was a modification of glycine bound to lysine in the identified peptide of huntingtin. Until now, the modification of huntingtin inclusions isolated from animal brains analyzed by LC–MS/MS directly have not been reported, but we found that K6 was ubiquitinated (1xGG [K5] modification; Fig. 2a) using nHTT inclusions obtained from HD190QG mouse brains. Taken together, the LC–MS/MS analysis revealed that nHTT was the major component of NIIs of HD190QG and ubiquitinated at K6. However, LC–MS/MS analysis did not show the sequence with expanded polyglutamine. Therefore, we performed amino acid analysis using the SDS-insoluble fraction containing NIIs.

### Glutamine significantly increased in the NII-rich fraction of HD190QG brains

**Fig. 3:**
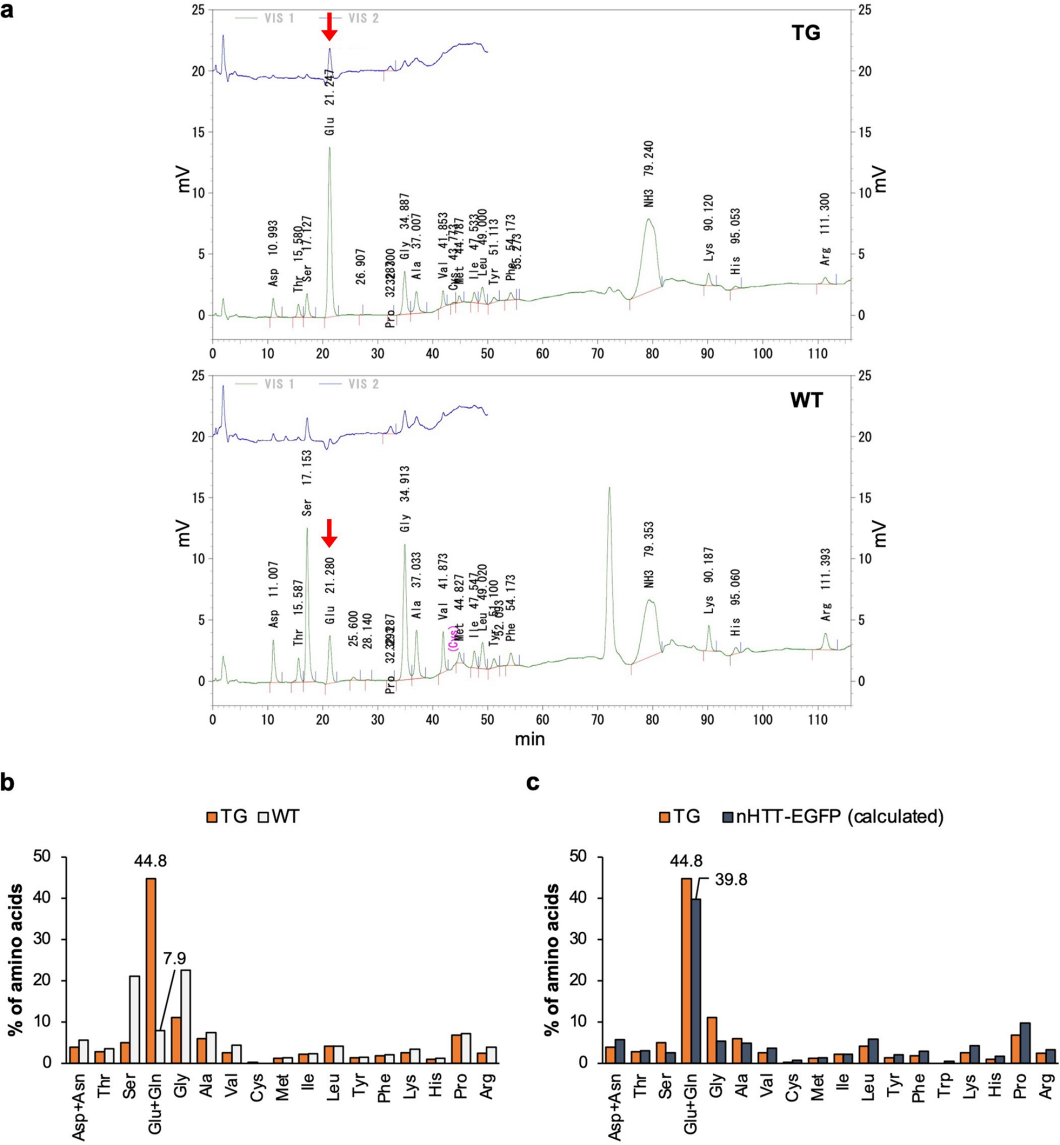
Glutamic acid significantly increased in the NIIs of HD190QG. **a** Chromatograms of amino acid analysis of the SDS-insoluble fraction of HD190QG (TG) and WT mouse brains. Glutamic acid is indicated by red arrows. **b** The percentage of amino acid numbers in the SDS-insoluble fraction of HD190QG (TG) and WT mice. **c** The percentage of amino acid numbers in the SDS-insoluble fraction of HD190QG mice (TG) resulted by amino acid analysis and the calculated percentage of amino acids in the sequence of nHTT-EGFP with expanded polyglutamine (190Q)

Since the SDS-insoluble fraction was confirmed to contain NIIs earlier (Fig. 1d), and hydrolysis with 6 M HCl would be performed to cleave the peptide bonds in proteins during the amino acid analysis, we did not treat the fraction with 70% FA. The chromatograms of amino acid analysis of HD190QG and WT showed that glutamic acid increased significantly in HD190QG (Fig. 3a). Glutamine and glutamic acid were analyzed together due to deamidation of glutamine during the hydrolysis process, but the total protein quantification was not affected because they have nearly equivalent molecular weights (146.13 g/mol for glutamine and 147.13 g/mol for glutamic acid, respectively). Asparagine and aspartic acid were also analyzed together due to deamidation of asparagine during the hydrolysis process. To confirm that glutamic acid or glutamine in TG increased significantly compared to WT, we calculated the percentage of each amino acid in the SDS-insoluble fraction of the HD190QG mice and found that the percentage of glutamine or glutamic acid in TG increased by about 5.7 times compared to WT (Fig. 3b). We also calculated the percentage of amino acid numbers in the sequence of nHTT-EGFP with expanded polyglutamine (190Q) and normal repeat size (21Q) expressed in HD190QG. As a result, the calculated percentage of glutamic acid and glutamine in 190Q increased about 2.7 times compared to 21Q (Fig. S1). We also compared the results of real amino acid content (TG) with the calculated amino acid content of HD190QG (nHTT-EGFP) and found that glutamine or glutamic acid of the NII-rich fraction of HD190QG brains increased significantly and is compatible with the calculated amino acid content of HD190QG (Fig. 3c). Taken together, we directly analyzed the amino acid composition of the NII-rich fractions of HD190QG brains and assume that this method could be useful for the analysis of NIIs of diseased human brains, which may have proteins with compositionally biased regions (CBRs).

### The NII-rich fraction was obtained from the NIID brain

**Fig. 4:**
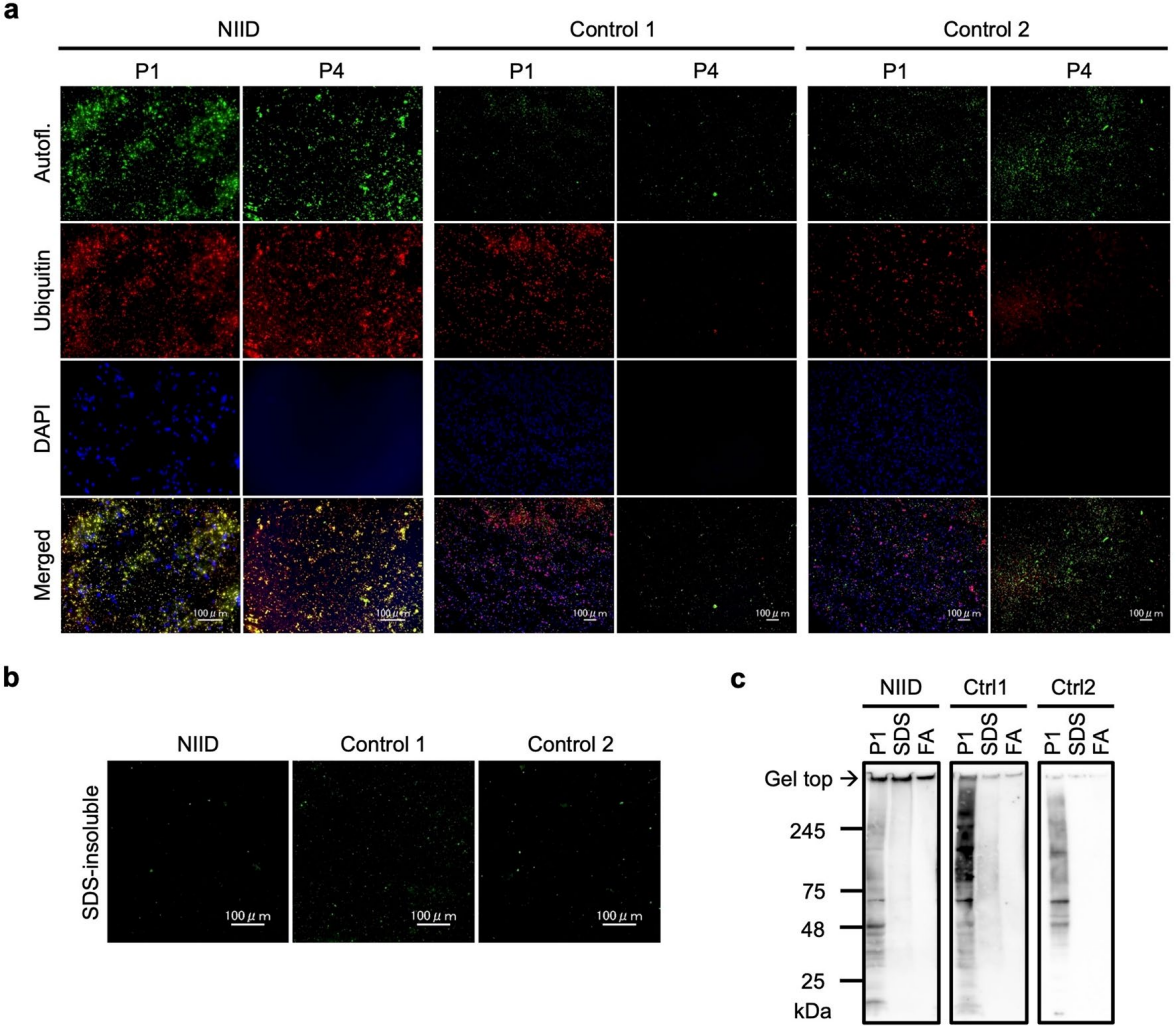
The NII-rich fraction was isolated from NIID and the control brains. **a** P1 and P4 fractions of NIID and the control brains were immunostained with the anti-ubiquitin antibody. Green signals indicate the autofluorescence of NIIs, and red signals indicate ubiquitin positive molecules. DAPI signals are observed as blue. Overlapping autofluorescence and ubiquitin positive signals indicating NIIs are observed as yellow in NIID. The autofluorescence observed in the control fractions could be lipofuscin, which does not overlap with ubiquitin positive signals. Scale bars = 100 µm. **b** Autofluorescence was observed in the SDS-insoluble fractions obtained from each brain sample. Scale bars = 100 µm. **c** P1, SDS-soluble (SDS) and FA-treated fraction (FA) were immunoblotted with the anti-ubiquitin antibody

The NIIs of the NIID brain and the control brains were isolated in the same way as the fractionation of NIIs from HD190QG mouse brains. The SDS-insoluble fractions were washed with 2% SDS/10 mM DTT solution after the first treatment to remove the remaining SDS-soluble proteins. Since the inclusions from the NIID brain used in this study are known to emit autofluorescence [2], we immunostained the P1 and P4 fractions with the anti-ubiquitin antibody to confirm that the autofluorescence is from the ubiquitinated inclusions (Fig. 4a). The autofluorescence observed in the NIID fractions overlapped with the anti-ubiquitin antibody-positive signals, indicating that the autofluorescence came from the NIIs. In the control fractions, autofluorescence was also observed, but the signals did not overlap with anti-ubiquitin antibody-positive signals. The autofluorescence observed in the control brains may be from lipofuscin, also known as age pigment, which accumulates during normal senescence and cannot be digested by the ubiquitin–proteasome system [33–35]. Autofluorescence was also observed in the SDS-insoluble fractions of all brain samples (Fig. 4b), which were later treated by 70% FA. Finally, immunoblot analysis with the anti-ubiquitin antibody revealed the gel-top staining, suggesting that NIIs were successfully isolated from the NIID brain (Fig. 4c). In order to investigate the composition of NIIs obtained from the NIID brain, the FA-treated fraction was subjected to LC–MS/MS.

### NIIs obtained from the NIID brain were rich in serine

**Fig. 5:**
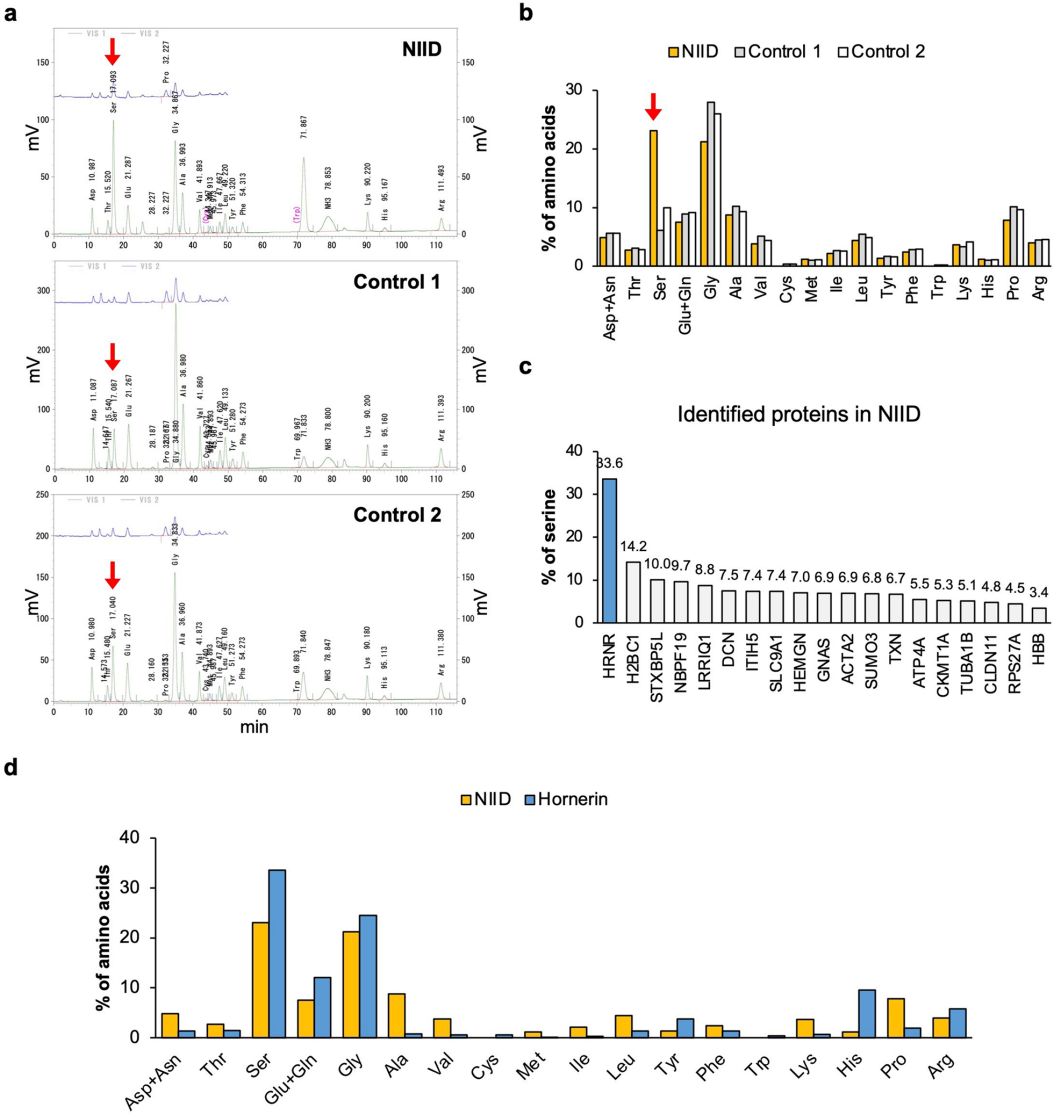
Serine significantly increased in NIIs obtained from the NIID brain. **a** Chromatograms of amino acid analysis using the SDS-insoluble fraction of NIID and the control brains. Serine is indicated by red arrows. **b** The calculated percentage of amino acid numbers in NIIs obtained from each brain. Serine is indicated by a red arrow. **c** The percentage of serine in the proteins identified in NIID. **d** The comparison between the percentage of amino acids of NIIs obtained from the NIID brain and the percentage of amino acids of hornerin protein

By performing LC–MS/MS with the trypsin-digested FA-treated fractions of the NIID and control brains, we observed 19 proteins that were specifically identified in NIIs from the NIID brain (Additional file 2, Table 1). We postulated that among the 19 proteins, including known nuclear proteins, any proteins that are related to either the formation of NIIs or the disease pathogenesis could be found. For amino acid analysis, the SDS-insoluble fractions of each brain sample were hydrolyzed by 4 N Methanesulfonic acid instead of 6 M HCl to prevent hydrolyzing tryptophan. In the NIID sample, we detected about 3.8 times more serine than in Control 1 and about 2.3 times more than in Control 2 samples, while all other amino acids were detected lesser amounts than in the controls (Fig. 5a, b). To figure out which protein in NIID was rich in serine, we calculated the percentage of amino acid numbers in the sequences of all the identified proteins and found that hornerin (HRNR) was the most serine-rich protein (Fig. 5c). We also confirmed that the pattern of amino acid contents in NIIs and hornerin are similar (Fig. 5d), indicating that hornerin might be one of the major components of the NIIs in NIID.

**Table 1:** The proteins in NIIs specifically identified in the NIID brains

Protein description	Accession
Small ubiquitin-related modifier 3 (SUMO3)	P55854
Thioredoxin (TXN)	P10599
Ubiquitin-40S ribosomal protein S27a (RPS27A)	P62979
Histone H2B type 1-A (H2BC1)	Q96A08
Claudin-11 (CLDN11)	O75508
Sodium/hydrogen exchanger 1 (SLC9A1)	P19634
Hemogen (HEMGN)	Q9BXL5
Hornerin (HRNR)	Q86YZ3
Syntaxin-binding protein 5-like (STXBP5L)	Q9Y2K9
Inter-alpha-trypsin inhibitor heavy chain (ITIH5)	Q86UX2
Leucine-rich repeat and IQ domain-containing protein 1 (LRRIQ1)	Q96JM4
Neuroblastoma breakpoint family member 19 (NBPF19)	A0A087WUL8
Actin, aortic smooth muscle (ACTA2)	P62736
Potassium-transporting ATPase alpha chain 1 (ATP4A)	P20648
Guanine nucleotide-binding protein G(s) subunit alpha isoforms XLas (GNAS)	Q5JWF2
Tubulin alpha-1B chain (TUBA1B)	P68363
Hemoglobin subunit beta (HBB)	P68871
Decorin (DCN)	P07585
Creatine kinase U-type, mitochondrial (CKMT1A)	P12532

### Hornerin is the major component of NIIs in NIID

**Fig. 6:**
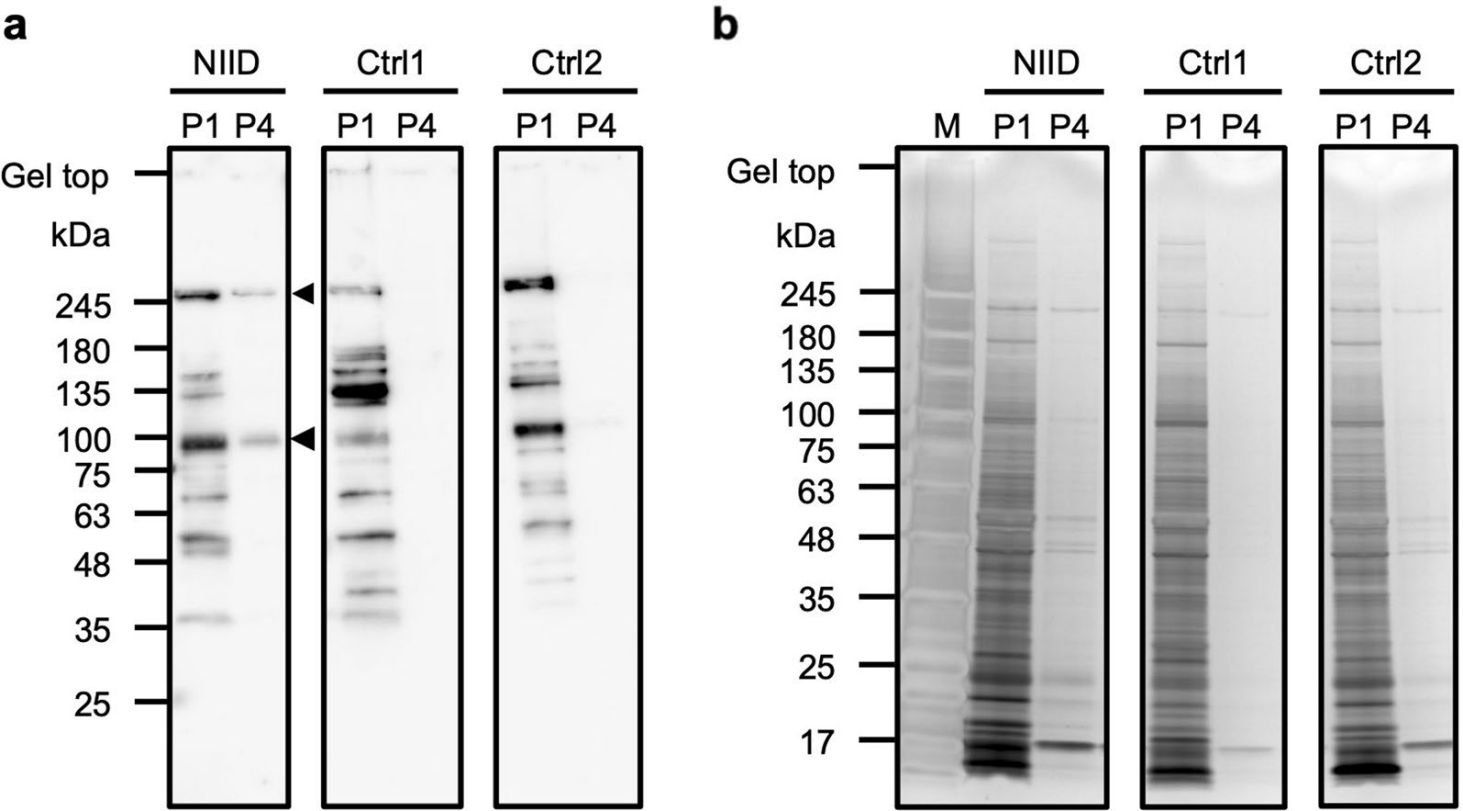
Hornerin was detected specifically in the NIID fractions. **a** Immunoblotting of hornerin with the P1 and P4 fractions. Arrowheads indicate anti-hornerin antibody-positive bands (245 kDa and 100 kDa). **b** Silver staining of the P1 and P4 fractions obtained from each brain sample

To confirm that hornerin is the major component of the NIIs, we performed Western blot using the P1 and P4 fractions of NIID. Anti-hornerin antibody-positive bands with the sizes of 100 kDa and 245 kDa were detected in P4 fraction (Fig. 6a). As the amount of protein seemed to be the same in each fraction of NIID, Control 1 and Control 2 (Fig. 6b), we hypothesized that the protein bands detected by Western blot would contain fragments of hornerin. To obtain and identify the peptides of interest stained by CBB, we performed in-gel tryptic digestion and LC–MS/MS and found that all the anti-hornerin antibody-positive bands contained the peptides of hornerin (Additional file 2, Additional file 4: Table S1 and Fig. S2). These results suggest that hornerin is one of the major components in the NIIs of NIID.

### A specific distribution of hornerin in the NIID brain was observed by MALDI-IMS

**Fig. 7:**
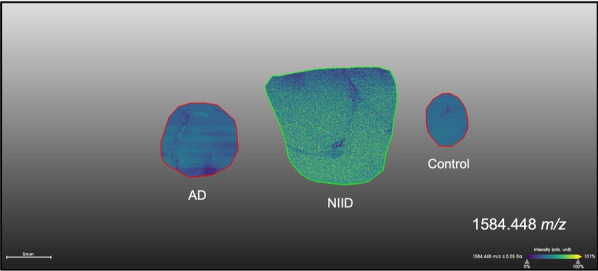
A specific distribution of the Hornerin peptide was detected by MALDI-IMS. MALDI-IMS was performed with brain sections of NIID, AD and a control. The distribution of the peptide at 1,584 m/z detected by LC–MS earlier was observed

Since LC–MS/MS revealed that hornerin was specifically identified in NIIs of NIID, we investigated the distribution of hornerin using an integrated analysis of MALDI-IMS and shotgun proteomics (LC-TIMS-MS/MS). MALDI-IMS is a technique for investigating the distribution of proteins and small molecules within biological systems, through the direct in situ analysis of tissue sections [22, 36]. The brain sections of NIID, AD and a control were used for MALDI-IMS and analyzed computationally. For LC-TIMS-MS/MS, we used 3 Gy matter samples and 2 white matter samples of NIID and other diseased brains, such as multiple system atrophy, progressive supranuclear palsy, AD and AD/cerebral amyloid angiopathy, as well as a control brain. The result of the integrated analyses of MALDI-IMS and LC-TIMS-MS/MS showed that the hornerin peptides were identified both in gray matter samples and white matter samples (Table S1 and Fig. S2), and a specific distribution in NIID was observed at 1,584 m/z (Fig. 7). This result corresponds to the LC–MS/MS result using the FA-treated fraction of NIIs (Additional file 2). In addition, more peptides were identified in the white matter samples compared to the gray matter samples.

### The localization of hornerin on NIIs was observed in the NIID brain by immunofluorescence

**Fig. 8:**
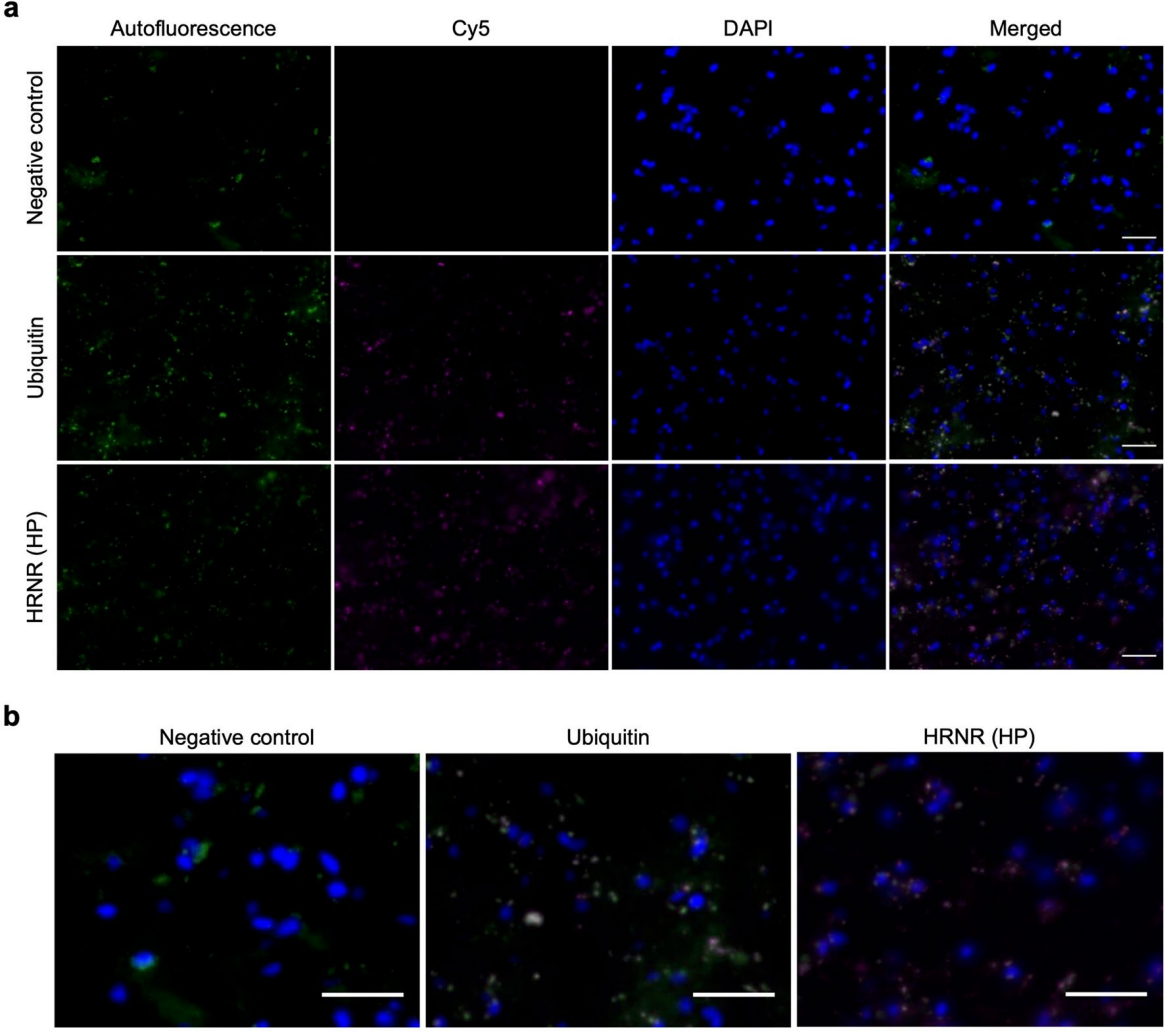
Hornerin was detected in the NIID brain sections by IF staining. **a** IF staining of the NIID brain sections with the anti-ubiquitin and anti-HRNR (HP) antibodies. Autofluorescent signals are shown in green, and the antibody-positive signals are shown in pink (Cy5). The cells are stained with DAPI (blue). Scale bars = 100 µm. **b** Enlarged images of the merged images in **a** Scale bars = 100 µm

In order to study the distribution of hornerin in the NIID brain, IF staining was performed on NIID sections, using a custom antibody termed anti-HRNR (HP), designed to detect human hornerin_968-1349_. Anti-ubiquitin antibody was used for a positive control, and immunostaining without any primary antibodies was performed as a negative control. All the NIID sections showed autofluorescence, and we found that anti-HRNR (HP) antibody-positive signals co-localized with the autofluorescent inclusions (Fig. 8).

### The Ser1008Thr variant in the *HRNR* gene was found in the NIID

**Fig. 9:**
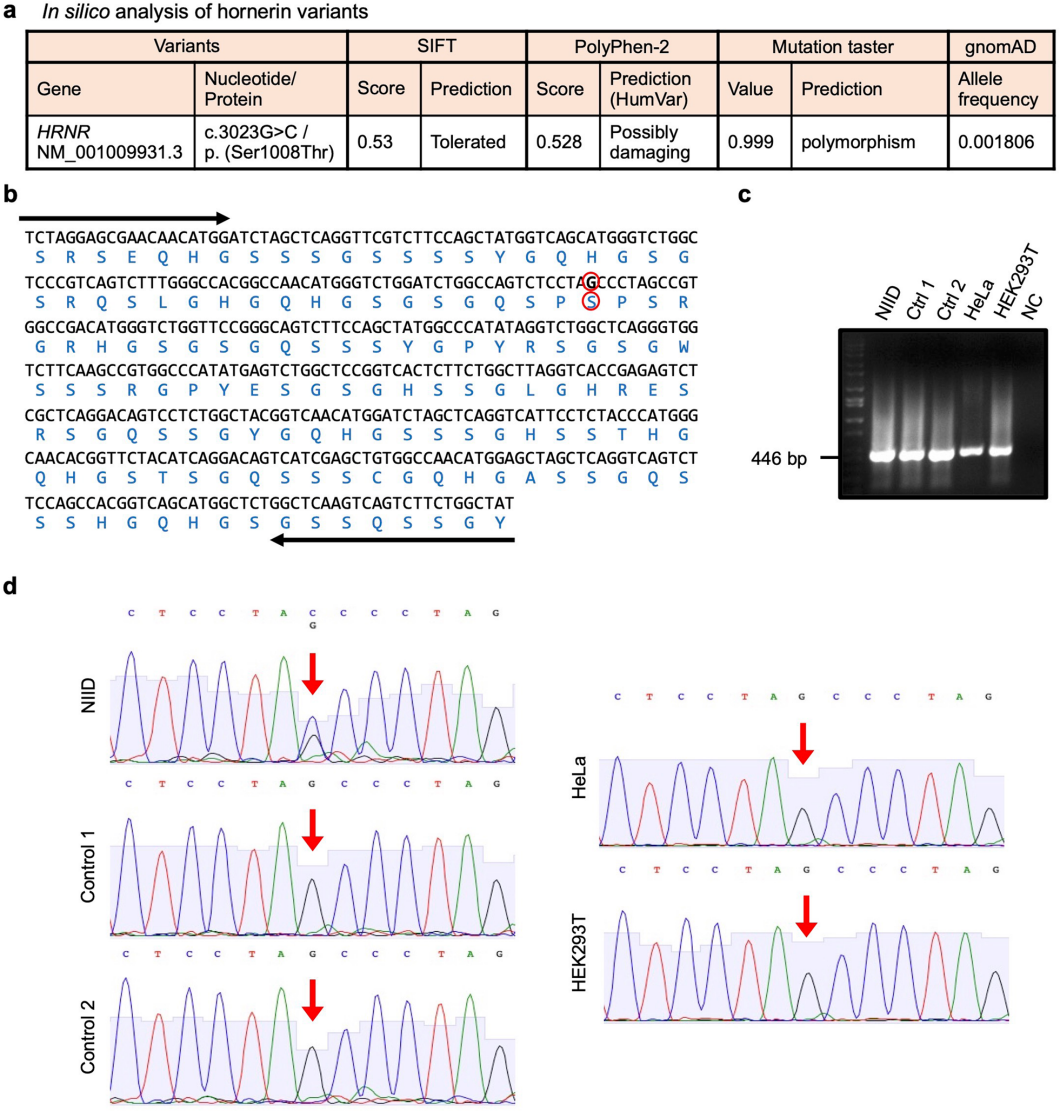
The analysis of hornerin variants. **a** A nonsynonymous variant, c.3023G > C/p. (Ser1008Thr), was found in the gene *HRNR* by the whole-exome sequencing. SIFT: The amino acid substitution is predicted. Scores range from 0 to 1. Damaging if the score is ≤ 0.05 and tolerated if the score is > 0.05 (https://sift.bii.a-star.edu.sg/www/SIFT_seq_submit2.html). PolyPhen-2: scores were evaluated from 0.000 (most probably benign) to 0.999 (most probably damaging). Mutation Taster: a value close to 1 indicates a high security of the prediction, and the alterations are classified as disease causing or polymorphisms. gnomAD: Genome Aggregation Database (http://gnomad.broadinstitute.org/). **b** DNA sequence of the PCR product used for variant detection. Oligonucleotide primers are shown by arrows. Amino acid translation is shown below the DNA sequence. The circled base represents the G3023C change in the mutant allele. The resulting amino acid Ser1008Thr change is represented by the circled amino acid. **c** PCR was performed with genomic DNAs of NIID, Control 1, Control 2 brains as well as HeLa and HEK293T cells. NC refers to negative control. **d** The G3023C variant in NIID was confirmed by DNA sequencing. The variant site is shown by red arrows

We next analyzed whole-exome sequencing and long-read sequencing data of genomic DNA extracted from the brain of the NIID patient in order to identify pathogenic variants. In this NIID case, the pathogenic GGC expansion was not identified in the *NOTCH2NLC* gene and other genes by long-read sequencing. The sequence analysis revealed a nonsynonymous variant NM_001009931.3:c.3023G > C, p. (Ser1008Thr) within exon 3 of the human *HRNR* gene with an amino acid change from serine to threonine at position 1008 (Fig. 9a). To confirm that this variant is heterozygous, DNA sequencing was performed with the PCR products derived from NIID and the control brains, as well as Hela and HEK293T cells. The primers were designed to amplify serine or threonine at position 1008 (Fig. 9b) and checked by PCR to ensure the primer efficiencies and also that they generated single bands of the predicted size (Fig. 9c). The PCR products were sequenced, and the G3023C variant was found heterozygously in NIID (Fig. 9d).

## Discussion

Until now, it has been difficult to analyze the protein components of NIIs directly due to the protein’s structural features and insolubility. In fact, polyglutamine was not identified by LC–MS/MS in insoluble fractions of HD model mouse brains (Additional file 1). This might be because protein cores are hard to solubilize, and thus cannot be ionized for LC–MS/MS. Therefore, we combined LC–MS/MS with amino acid analysis in order to search for candidate pathogenic proteins of NIID [2]. Because amino acid analysis showed a high content of serine in the NII-rich fraction, and hornerin was found to be serine-rich, we assumed that hornerin could be a main component of the NIIs in our patient. Based on the unique distribution of hornerin in NIID and the anti-hornerin antibody-positive signals in the NIIs, as shown by MALDI-IMS and IF staining respectively, we suggest that hornerin is the main component of the NIIs being involved in the pathogenic cascade of the disease.

Human hornerin is a 245 kDa protein which shares structural features with S100 fused-type proteins, particularly with profilaggrin. In mice, hornerin was found to be expressed in cornifying stratified epithelium, including the epidermis, tongue, esophagus, and forestomach. In the epidermis, hornerin co-localized with profilaggrin in keratohyalin granules, indicating that hornerin plays roles similar to those of profilaggrin in the cornifying epithelium [37, 38]. The expression of hornerin was also observed in mouse neurons (Allen mouse brain atlas, https://mouse.brain-map.org/gene/show/44565). In humans, hornerin was detected in regenerating skin after wounding and in psoriatic skin [37]. Hornerin was also expressed abundantly in healthy human skin with unusual biochemical characteristics including forming and auto-degrading properties of aggregates [39]. The roles of hornerin in tumor progression or vascularity in several cancers including breast cancer [40, 41], hepatocellular carcinoma [42] and pancreatic ductal carcinoma [43] have been investigated. Although the expression of hornerin has been observed in many tissues, its expression in human brain sections has not been reported, and little is known about the roles of hornerin in neurodegenerative diseases.

Hornerin is an IDP with LCRs or CBRs, which contains 95% tandem quasi-repeating glycine- and serine-rich domains. Unlike globular proteins, IDPs contain a low quantity of hydrophobic amino acids, stabilizing the protein structures, but are rich in polar and charged amino acids, interacting favorably with water [44, 45]. This low sequence complexity of IDPs may affect protein misfolding and aggregation in neurodegenerative diseases such as AD and PD. In fact, many disease-related IDPs, including ⍺-syn and tau, are found to contain LCRs or CBRs in their sequences. It is challenging to identify the components of protein aggregates by LC–MS/MS, which we tried using to identify polyglutamine in NIIs of HD190QG model mice. However, it could not identify expanded polyglutamine of huntingtin, which has low sequence complexity. Therefore, we performed amino acid analysis and successfully confirmed that glutamine increased significantly in NIIs of HD190QG (Fig. 3). By combining LC–MS/MS and amino acid analysis with additional bioinformatic analyses, we found a significantly increased serine content in the NII-rich fraction obtained from the NIID brain, later identified as hornerin deposits. Thus, it is important to analyze either the protein aggregates or the NII-rich fraction of those diseases at amino acid level. In addition, since the first discovery of RAN translation in SCA8 and myotonic dystrophy type 1, RAN translated products have been suggested in a growing number of diseases including fragile X tremor ataxia syndrome, HD and SCA31 [46]. RAN translation could produce the proteins with CBRs. Our methods should be used to investigate protein aggregates in those diseases.

Since hornerin was found in NIIs, we investigated whether this protein has nuclear localization signals (NLSs) via several computational NLS prediction tools: cNLS Mapper [47], NLStradamus [48] and seqNLS [49]. However, no NLS was predicted in the sequence of hornerin, and how hornerin could be imported to the nucleus without NLS remains unclear. A previous study reported that N-terminal spacer domain peptides of profilaggrin, which shares features with hornerin, are transported to the nucleus [50]. There are also some proteins that do not have NLS but can be transported to the nucleus in their NLS-independent manner [51]. Hornerin might be one of these proteins that can be transported to the nucleus NLS-independently. Further studies are necessary to investigate the nuclear entry mechanism of hornerin.

The disease-related IDPs also undergo post-translational modifications (PTMs) such as phosphorylation or ubiquitination, which may enhance their aggregation property [52, 53]. In our LC–MS/MS analysis results, ubiquitination of hornerin was not identified. This might be because ubiquitinated fragments were not included in the identified peptides. However, carbamylation at N-termini was found as a PTM that hornerin might undergo (Additional file 2). Since carbamylation of lysine residues and protein N-termini, which is a hallmark of aging, can be artificially introduced during sample preparation with urea [54], the direct effect of carbamylation on the properties of hornerin remains unclear. Although we could not detect other modifications in our study, hornerin fragments were identified in the Western blot. This fragmentation could enhance the aggregation property of hornerin.

In this NIID case, the pathogenic variants of known NIID genes *NOTCH2NLC* and CAG repeats were not identified. Our protein chemical analysis clearly showed hornerin is the major component of NIIs of this NIID. However, it is still possible that an unidentified gene with repeat expansion producing polyserine is the pathogenic gene of this NIID, and hornerin is recruited to the polyserine inclusion. Further revised long-read sequencing to detect repeat expansion expressing polyserine is necessary. Although we found LRRK2:NM_198578:exon38:c.T5606C:p.M1869T [55], this variant is responsible for adult-onset PD, therefore, it is unrelated to this NIID. A variant (G3023C) within the human *HRNR* gene with an amino acid change (Ser1008Thr) was found via whole-exome sequencing of this NIID (Additional file 3). However, using gnomAD browser [56], we found that the allele frequency of this variant is 0.001806 (Fig. 9a). This means that this variant in *HRNR* occurs 1 in 300 Europeans, while other known pathogenic gene variants such as the Gly2019Ser variant of *LRRK2* in PD (1 in 2,400 Europeans) or the Arg521Cys variant of FUS in amyotrophic lateral sclerosis (1 in 25,000 Europeans) are much less frequent. Most of the heterozygous variants, which cause serious symptoms from early childhood, are de novo. However, in terms of frequency, it is hard to think that this Ser1008Thr variant is de novo. Since the in silico prediction also revealed that the variant is non-pathogenic (Fig. 9a), this variant itself is unlikely to be responsible for NIID. Although more analyses are certainly needed to determine the genetic role of hornerin variants, a hornerin cascade should be recognized as a pathology of this disease. In this regard, the variant analysis of genes related to hornerin binding proteins should be investigated.

## Supplementary Information

**Additional file 1**: LC-MS/MS result of HD190QG mouse brains.

**Additional file 2**: LC-MS/MS result of NIID and control brains.

**Additional file 3**: Whole exome sequencing data.

**Additional file 4.**: **Table S1**. The identified hornerin peptides by LC-MS/MS and LC-TIMS-MS/MS. **Figure S1**. The calculated percentage of amino acids in the sequence of nHTT-EGFP. **Figure S2**. The hornerin peptides identified by LC-MS/MS and LC-TIMS-MS/MS.
