# Supplementary material for: Hornerin deposits in neuronal intranuclear inclusion disease: direct identification of proteins with compositionally biased regions in inclusions
Source: Acta Neuropathol Commun. 2022 Mar 4;10:28. doi: 10.1186/s40478-022-01333-8 (PMC8895595; doi:10.1186/s40478-022-01333-8)
Supplement: Supplementary file 4 — Additional file 4.: Table S1. The identified hornerin peptides by LC-MS/MS and LC-TIMS-MS/MS. Figure S1. The calculated percentage of amino acids in the sequence of nHTT-EGFP. Figure S2. The hornerin peptides identified by LC-MS/MS and LC-TIMS-MS/MS. [file 40478_2022_1333_MOESM4_ESM.pdf]

Supplementary Information

Hornerin deposits in neuronal intranuclear inclusion disease: Direct identification of proteins with compositionally biased regions in inclusions

Hongsun Park, Tomoyuki Yamanaka, Yumiko Toyama, Atsushi Fujita, Hiroshi Doi, Takashi Nirasawa, Shigeo Murayama, Naomichi Matsumoto, Tomomi Shimogori, Masaya Ikegawa, Nobuyuki Nukina*

Separate excel files

Supplementary dataset 1

Supplementary dataset 2

Supplementary dataset 3

Supplementary Table

Table S1. The identified hornerin peptides by LC-MS/MS and LC-TIMS-MS/MS.

The peptides at 1,584 m/z were highlighted. See figure S2 for a map.

Analysis	#	Samples	Sequence	Number of repeats
**LC-MS/MS**	**1**	FA fraction	GPYESGSGHSSGLGHR	3
	**2**	P4 245 kDa	GPYESGSGHSSGLGHQESR	1
	**3**		GPYESGSGHSSGLGHR	3
	**4**		GSGSGQSPSSGQHGTGFGR	1
	**5**		HGSGSGHSSSYGQHGSGSGWSSSSGR	3
	**6**		HGSGSGQSSSYGPYR	1
	**7**		HGSGSGQSSSYSPYGSGSGWSSSR	2
	**8**		QSLGHGQHGSGSGQSPSPSR	4
	**9**	P4 100 kDa	QSLGHGQHGSGSGQSPSPSR	4
	**10**		GSGSGQSPSYGR	1
	**11**		SGSGWSSSR	4
**LC-TIMS-MS/MS**	**12**	Gray matter 1	QGSGSGQSPGHGQ	4
	**13**	Gray matter 2	YGQQGSGSGQSPSR	3
	**14**	White matter 1	SSSGSSSSYGQHGSGS	5
	**15**		QSSSYGPHGYGSGR	1
	**16**		GPYESGSGHSSGLGHQESR	1
	**17**		YGQQGSGSGQSPSR	3
	**18**		GPYESGSGHSSGLGHR	3
	**19**	White matter 2	SSSGSSSSYGQHGSGSR	5
	**20**		QSSSYGQHESASR	1
	**21**		GSGSGQSPSSGQHGTGFGR	1
	**22**		HGSGSGQSPSPSR	5
	**23**		QSSSYGPHGYGSGR	1
	**24**		GPYESGSGHSSGLGHQESR	1
	**25**		YGQQGSGSGQSPSR	3
	**26**		SGSGQSSGYSQHGSGSSHSSGYR	1
	**27**		SEQHGSSSGSSSSYGQHGSGSR	1
	**28**		GPYESGSGHSSGLGHR	3
	**29**		SSSRGPYESR	4
	**30**		HGSGSGQSSSYSPYGSGSGWSSSR	2
	**31**		GEQHGSSSGSSSSYGQHGSGSR	1
	**32**		HGSGSGQSSSYGPYGSGSGWSSSR	1
	**33**		HGAGSGQSLSHGR	1

Supplementary Figures

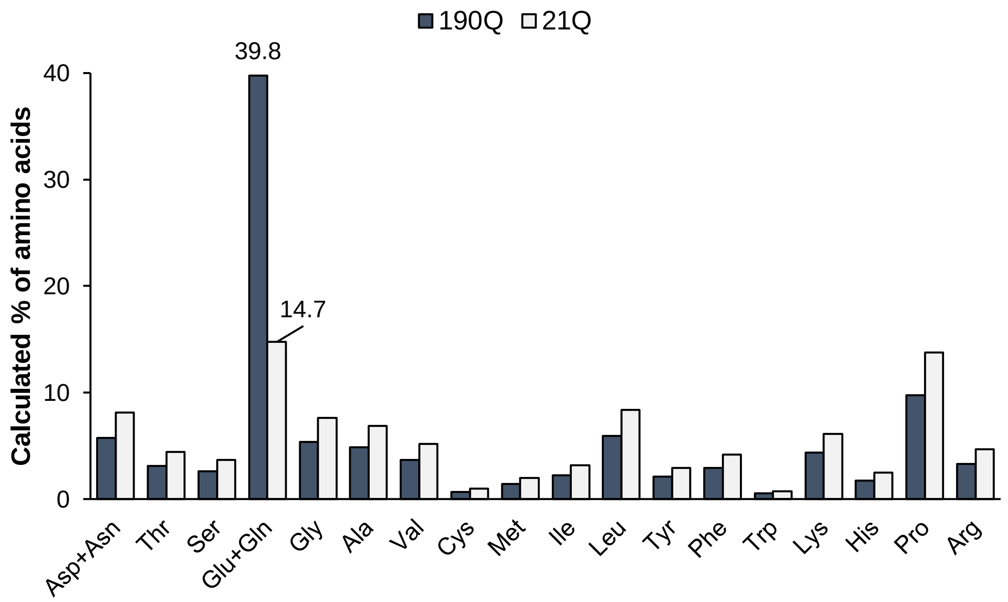

Fig. S1 The calculated percentage of amino acids in the sequence of nHTT-EGFP

The calculated percentage of amino acid numbers in the sequences of nHTT-EGFP with expanded polyglutamine (190Q) and normal repeat size (21Q) expressed in HD190QG mice.

```
MPKLLQGVIT VIDVFYQYAT QHGEYDTLNK AELKELLENE FHQILKNPND PDTVDIILQS LDRDHNKKVD FTEYLLMIFK LVQARNKIIG KDYCQVSGSK 100

LRDDTHQHQE EQEETEKEEN KRQESSFSHS SWSAGENDSY SRNVRGSLKP GTESISRRLS FQRDFSGQHN SYSGQSSSYG EQNSDSHQSS GRGQCGSGSG 200

QSPNYGQHGS GSGQSSSNDT HGSGSGQSSG FSQHKSSSGQ SSGYSQHGSG SGHSSGYGQH GSRSGQSSRG ERHRSSSGSS SSYGQHGSGS RQSLGHGRQG 300
                                                                                 #14, 19
SGSRQSPSHV RHGSGSGHSS SHGQHGSGSS YSYSRGHYES GSGQTSGFGQ HESGSGQSSG YSKHGSGSGH SSSQGQHGST SGQASSSGQH GSSSRQSSSY 400
                                                                                                    #20
GQHESASRHS SGRGQHSSGS GQSPGHGQRG SGSGQSPSSG QHGTGFGRSS SSGPYVSGSG YSSGFGHHES SSEHSSGYTQ HGSGSGHSSG HGQHGSRSGQ 500
                               #4, 21
SSRGERQGSS AGSSSSYGQH GSGSRQSLGH SRHGSGSGQS PSPSRGRHES GSRQSSSYGP HGYGSGRSSS RGPYESGSGH SSGLGHQESR SGQSSGYGQH 600
                                  #22                #15, 23          #2, 16, 24
GSSSGHSSTH GQHGSTSGQS SSCGQHGATS GQSSSHGQHG SGSSQSSRYG QQGSGSGQSP SRGRHGSDFG HSSSYGQHGS GSGWSSSNGP HGSVSGQSSG 700
                                                 #13, 17, 25
FGHKSGSGQS SGYSQHGSGS SHSSGYRKHG SRSGQSSRSE QHGSSSGLSS SYGQHGSGSH QSSGHGRQGS GSGHSPSRVR HGSSSGHSSS HGQHGSGTSC 800
   #26
SSSCGHYESG SGQASGFGQH ESGSGQGYSQ HGSASGHFSS QGRHGSTSGQ SSSSGQHDSS SGQSSSYGQH ESASHHASGR GRHGSGSGQS PGHGQRGSGS 900
                                                                                                     #10
GQSPSYGRHG SGSGRSSSSG RHGSGSGQSS GFGHKSSSGQ SSGYTQHGSG SGHSSSYEQH GSRSGQSSRS EQHGSSSGSS SSYGQHGSGS RQSLGHGQHG 1000
                                                                      #27   #14, 29           #8, 9   #22
SGSGQSPSPS RGRHGSGSGQ SSSYGPYRSG SGWSSSRGPY ESGSGHSSGL GHRESRSGQS SGYGQHGSSS GHSSTHGQHG STSGQSSSCG QHGASSGQSS 1100
 S1008T variant  #6         #11        #1, 3, 18, 28
SHGQHGSGSS QSSGYGRQGS GSGQSPGHGQ RGSGSRQSPS YGRHGSGSGR SSSSGQHGSG LGESSGFGHH ESSSGQSSSY SQHGSGSGHS SGYGQHGSRS 1200
                  #12
GQSSRGERHG SSSGSSSHYG QHGSGSRQSS GHGRQGSGSG HSPSRGRHGS GLGHSSSHGQ HGSGSGRSSS RGPYESRSGH SSVFGQHESG SGHSSAYSQH 1300
                                                                      #29
GSGSGHFCSQ GQHGSTSGQS STFDQEGSST GQSSSYGHRG SGSSQSSGYG RHGAGSGQSP SRGRHGSGSG HSSSYGQHGS GSGWSSSSGR HGSGSGQSSG 1400
                                                                     #5
FGHHESSSWQ SSGCTQHGSG SGHSSSYEQH GSRSGQSSRG ERHGSSSGSS SSYGQHGSGS RQSLGHGQHG SGSGQSPSPS RGRHGSGSGQ SSSYSPYGSG 1500
                                                #14, 19          #8, 9   #22            #7, 30          #11
SGWSSSRGPY ESGSSHSSGL GHRESRSGQS SGYGQHGSSS GHSSTHGQHG STSGQSSSCG QHGASSGQSS SHGQHGSGSS QSSGYGRQGS GSGQSPGHGQ 1600
                                                                                                  #12
RGSGSRQSPS YGRHGSGSGR SSSSGQHGSG LGESSGFGHH ESSSGQSSSY SQHGSGSGHS SGYGQHGSRS GQSSRGERHG SSSRSSSRYG QHGSGSRQSS 1700

GHGRQGSGSG QSPSRGRHGS GLGHSSSHGQ HGSGSGRSSS RGPYESRSGH SSVFGQHESG SGHSSAYSQH GSGSGHFCSQ GQHGSTSGQS STFDQEGSST 1800
                                      #29
GQSSSHGQHG SGSSQSSSYG QQGSGSGQSP SRGRHGSGSG HSSSYGQHGS GSGWSSSSGR HGSGSGQSSG FGHHESSSWQ SSGYTQHGSG SGHSSSYEQH 1900
                   #13, 17, 25      #5, 20
GSRSGQSSRG EQHGSSSGSS SSYGQHGSGS RQSLGHGQHG SGSGQSPSPS RGRHGSGSGQ SSSYGPYGSG SGWSSSRGPY ESGSGHSSGL GHRESRSGQS 2000
          #31    #14, 19           #8, 9   #22             #32          #11       #1, 3, 18, 28
SGYGQHGSSS GHSSTHGQHG SASGQSSSCG QHGASSGQSS SHGQHGSGSS QSSGYGRQGS GSGQSPGHGQ RGSGSRQSPS YGRHGSGSGR SSSSGQHGPG 2100
                                                          #12
LGESSGFGHH ESSSGQSSSY SQHGSGSGHS SGYGQHGSRS GQSSRGERHG SSSGSSSRYG QHGSGSRQSS GHGRQGSGSG HSPSRGRHGS GSGHSSSHGQ 2200

HGSGSGRSSS RGPYESRSGH SSVFGQHESG SGHSSAYSQH GSGSGHFCSQ GQHGSTSGQS STFDQEGSST GQSSSHGQHG SGSSQSSSYG QQGSGSGQSP 2300
   #29                                                                                           #13, 17, 25
SRGRHGSGSG HSSSYGQHGS GSGWSSSSGR HGSGSGQSSG FGHHESSSWQ SSGYTQHGSG SGHSSSYEQH GSRSGQSSRG ERHGSSSGSS SSYGQHGSGS 2400
   #5                                                                                            #14, 19
RQSLGHGQHG SGSGQSPSPS RGRHGSGSGQ SSSYSPYGSG SGWSSSRGPY ESGSGHSSGL GHRESRSGQS SGYGQHGSSS GHSSTHGQHG STSGQSSSCG 2500
 #8, 9  #22             #7, 30        #11       #1, 3, 18, 28
QHGASSGQSS SHGQHGSGSS QSSGYGRQGS GSGQSPGHGQ RGSGSRQSPS YGRHGSGSGR SSSSGQHGSG LGESSGFGHH ESSSGQSSSY SQHGSGSGHS 2600
                       #12
SGYGQHGSRS GQSSRGERHG SSSGSSSHYG QHGSGSRQSS GHGRQGSGSG QSPSRGRHGS GLGHSSSHGQ HGSGSGRSSS RGPYESRLGH SSVFGQHESG 2700
                                                                                 #29
SGHSSAYSQH GSGSGHFCSQ GQHGSTSGQS STFDQEGSST GQSSSYGHRG SGSSQSSGYG RHGAGSGQSL SHGRHGSGSG QSSSYGQHGS GSGQSSGYSQ 2800
                                                                    #33
HGSGSGQDGY SYCKGGSNHD GGSSGSYFLS FPSSTSPYEY VQEQRCYFYQ                                                 2850
```

Fig. S2 The hornerin peptides identified by LC-MS/MS and LC-TIMS-MS/MS

Every repeating peptide sequence is indicated with a particular color. The peptide numbers are listed in table S1.
